# Supplementary material for: An afferent white matter pathway from the pulvinar to the amygdala facilitates fear recognition
Source: eLife. 2019 Jan 16;8:e40766. doi: 10.7554/eLife.40766 (PMC6335057; doi:10.7554/eLife.40766)
Supplement: Transparent reporting form [file elife-40766-transrepform.docx]

***eLife’s* transparent reporting form**

We encourage authors to provide detailed information *within their submission* to facilitate the interpretation and replication of experiments. Authors can upload supporting documentation to indicate the use of appropriate reporting guidelines for health-related research (see [EQUATOR Network](http://www.equator-network.org/%20)), life science research (see the [BioSharing Information Resource](https://biosharing.org/)), or the [ARRIVE guidelines](http://www.plosbiology.org/article/info:doi/10.1371/journal.pbio.1000412) for reporting work involving animal research. Where applicable, authors should refer to any relevant reporting standards documents in this form.

If you have any questions, please consult our Journal Policies and/or contact us: [editorial@elifesciences.org](mailto:editorial@elifesciences.org).

**Sample-size estimation**

- You should state whether an appropriate sample size was computed when the study was being designed
- You should state the statistical method of sample size computation and any required assumptions
- If no explicit power analysis was used, you should describe how you decided what sample (replicate) size (number) to use

Please outline where this information can be found within the submission (e.g., sections or figure legends), or explain why this information doesn’t apply to your submission:

No explicit power analysis was performed but we outline the sample sizes of previous similar studies (see lines 54-59) and it is clear that our current sample size of 622 participants is approximately 20 times larger than previous studies.

**Replicates**

- You should report how often each experiment was performed
- You should include a definition of biological versus technical replication
- The data obtained should be provided and sufficient information should be provided to indicate the number of independent biological and/or technical replicates
- If you encountered any outliers, you should describe how these were handled
- Criteria for exclusion/inclusion of data should be clearly stated
- High-throughput sequence data should be uploaded before submission, with a private link for reviewers provided (these are available from both GEO and ArrayExpress)

Please outline where this information can be found within the submission (e.g., sections or figure legends), or explain why this information doesn’t apply to your submission:

All data used in this manuscript is publicly available via the Human Connectome Project (data availability is described in lines 695-700).

Outlier information is explained for every analysis (see lines 141, 200, 298, and 313). All statistics with outliers included are reported in the **Supplementary Materials**.

Initial inclusion criteria for selecting participants from the S900 HCP release is detailed in lines 464-479. Inclusion criteria for the DCM analysis are detailed in lines 656-671. Inclusion criteria for the DCM analysis on the subsample of unrelated (i.e. non-sibling) participants are detailed in line 3 of the supplementary materials and lines 475-479 in the main manuscript.

**Statistical reporting**

- Statistical analysis methods should be described and justified
- Raw data should be presented in figures whenever informative to do so (typically when N per group is less than 10)
- For each experiment, you should identify the statistical tests used, exact values of N, definitions of center, methods of multiple test correction, and dispersion and precision measures (e.g., mean, median, SD, SEM, confidence intervals; and, for the major substantive results, a measure of effect size (e.g., Pearson's r, Cohen's d)
- Report exact p-values wherever possible alongside the summary statistics and 95% confidence intervals. These should be reported for all key questions and not only when the p-value is less than 0.05.

Please outline where this information can be found within the submission (e.g., sections or figure legends), or explain why this information doesn’t apply to your submission:

All statistical analysis methods are explicitly stated (see the **dMRI analysis** and **fMRI analysis** sections of the **Methods**). Details can also be found throughout the **Results** sections and a full review of all statistical tests conducted is included in the tables of the **Supplementary Materials**. Exact *p* values are reported, as are effect sizes (partial eta squared) for repeated-measures ANOVAs, means and standard deviations, and sample size (see **Results** sections).

Raw data is represented via dot plots in **Fig. 1**, **Fig. 2**, **Fig. 5,** and **Fig. 6**.

(For large datasets, or papers with a very large number of statistical tests, you may upload a single table file with tests, Ns, etc., with reference to sections in the manuscript.)

**Group allocation**

- Indicate how samples were allocated into experimental groups (in the case of clinical studies, please specify allocation to treatment method); if randomization was used, please also state if restricted randomization was applied
- Indicate if masking was used during group allocation, data collection and/or data analysis

Please outline where this information can be found within the submission (e.g., sections or figure legends), or explain why this information doesn’t apply to your submission:

N/A – no group allocation

**Additional data files (“source data”)**

- We encourage you to upload relevant additional data files, such as numerical data that are represented as a graph in a figure, or as a summary table
- Where provided, these should be in the most useful format, and they can be uploaded as “Source data” files linked to a main figure or table
- Include model definition files including the full list of parameters used
- Include code used for data analysis (e.g., R, MatLab)
- Avoid stating that data files are “available upon request”

Please indicate the figures or tables for which source data files have been provided:

All analysis code is publicly available on the corresponding author’s GitHub, the details of which are described in the **Code Availability** section (lines 691-694).
